# Guernsey Breeders’ Association

**Published:** 1895-04

**Authors:** 


### SELECTION.

---

### GUERNSEY BREEDERS' ASSOCIATION.

The Guernsey Breeders' Association held their annual meeting at Philadelphia, on January 28. The President, Mr. Silas Betts, in his annual address made the following statement referring to tuberculosis among bovines, that he was convinced long ago that many of the veterinary profession had made a mistake of taking the effect for the cause; that the disease can never be exterminated by the slaughtering of cattle and the use of tuberculin; that the infection was from the city to the country, not from the country to the city; that biology does not justify the veterinarians in their statement that the thoroughbred and the closebred are most likely to be affected. He said that we should have State Boards of Health for the inspection of premises, but the dairymen should have chief charge of their own property and premises.

Dr. Samuel G. Dixon, of the Academy of Natural Sciences, referring to the danger from the disease in milk, that the people were unnecessarily alarmed regarding the danger of contracting tuberculosis from cows' milk, and that the physicians in Pennsylvania have much difficulty in persuading their patients to use cows' milk liberally without more or less nervous tension on the part of the sick ; further, that a large majority of the bacteriologists of the world have searched most diligently for the tubercle bacilli in the milk of cows suffering from consumption, and yet have only found the germs in that taken from animals whose udders were affected, and that in incipient tuberculosis these glands are rarely affected, and that thus the danger is greatly lessened. In answer to the questions regarding the compulsory use of tuberculin as a diagnostic agent for tuberculous cattle, he replied: "I can answer most emphatically that I believe and know it would be a grave mistake; while admitting the value of tuberculin as a useful diagnostic agent, that at the same time it is a powerful poison that is surrounded by possible dangers." He claimed to have produced immunity in animals in laboratory experiments by tuberculin, and produced a predisposition to the disease by the use of larger doses of the same material. He further stated that he believed by its general and indiscriminate use in cattle as a diagnostic agent a comparatively harmless tuberculosis would often be fanned into a condition that would render innocuous milk poisonous to the consumers. He thought as a diagnostic agent it should be only used in conspicuous cases.

A letter from Freudenreish, of Berne, Minister of Agriculture of Switzerland, concerning the use of tuberculin, said that its use was permitted by the government he represented for detecting tuberculous cattle. Quoting Professor Guillehean, of the Berne Veterinary School, as unfavorable to its use, on the grounds that while it detects it generalizes the disease in two or three weeks, where the disease might have been latent for years; further, that he believes cattle are always contaminated by the servants or attendants.

Dr. W. L. Zuill, of Philadelphia, at the same meeting, referring to the question of tuberculosis and the use of tuberculin as a diagnostic agent, said: "There is no question but that the danger scare which has been given to the public concerning the use of milk has been greatly exaggerated. Statistical experimentations have proven this over and over again, and they conclusively show that the milk from a cow with chronic or encysted tubercular centres is not necessarily dangerous or unfit for food, as it may contain neither the spore nor the germ of the disease, and that tuberculin is far from being the infallible diagnostic agent that some claim for it. The first investigation in the United States to determine the diagnostic value of this medicament in tubercular disease of cattle was made by a committee of the veterinary faculty of the University of Pennsylvania, of which I was chairman. The conclusions reached by that committee have been corroborated by every similar investigation since that time; they all show that tuberculin will not give a reaction in every case of tubercular disease, and that it will give a reaction where there is no tuberculosis in cases of chronic pneumonia, actinomycosis, sarcoma, contagious pleuro-pneumonia, etc. Therefore it is not reliable in diagnosis.

"In addition to its unreliability it is positively dangerous, inasmuch as it may change the milk of a dairy which was perfectly pure and harmless into a noxious product, by changing a chronic and encysted tubercular centre into an acute miliary tuberculosis, thus filling the milk with germs and spores, rendering it dangerous, virulent and unfit for food.

"This form of the disease is probably never seen in cattle, except as the result of a tuberculin injection, as the natural course of the disease is always chronic, and it is only when it has progressed until there is a general infection, including a tubercular mammitis, that the milk becomes dangerous. This condition may easily be recognized by the physical condition of the udder, or by bacteriological examination of the milk.

"We, as veterinarians, have no right to jeopardize a man's property by injecting his cattle with tuberculin, nor to slaughter his animals in an endeavor to prove that they produce diseased milk, as the finding of tubercular lesions is not proof of this fact. Does the law allow a building inspector to tear down and destroy a man's house in order to find out whether or not it is properly built, and then ask him to rebuild it? Then why should the law allow a dairy inspector to kill a cow in order to prove that her milk product was unwholesome. Would it not be more reasonable, more logical, more in accord with the dictates of common sense to first examine that product microscopically and chemically, and thereby prove that it is unfit for food and dangerous to human life. Such an examination can be made with less loss of time, with more certainty and less expense, and when made has a more definite and fixed value than any test to which the animal could be subjected.

"It is agreed that milk is not likely to contain bacilli unless the udder is affected with the disease, and the percentage of cows with tubercular mammitis is very small. The most recent experiments go to prove that milk containing tubercular bacilli when diluted with other milk to the extent of one part to fifty is rendered innocuous as an article of food, or at least reduces the danger to a minimum.

"There are hundreds of human beings who would be alive to-day, so far as the disease of tuberculosis (which they had) is concerned, if, in the excitement following the announcement of Koch's cure, they had not submitted themselves to the treatment. The inoculation broke down the chronic and encysted centres in their lungs, and they died within a few months after receiving the injection.

"My position is this: I cannot, do not, and will not indorse the indiscriminate use of tuberculin as an agent for diagnosing tuberculosis in dairy cattle. Every new report we get of the general arbitrary use of this substance proves it unreliable. It is not used by law in any country in the Old World, as far as I have been able to learn."

[At the Veterinary Section of the Eighth Annual Congress of Hygiene and Demography, held at Budapest, the subject of tuberculosis was considered by Professors Bang, Hess, Siedamgrotzky, Nocard, Perroncito, and Thomassen especially as to the diagnostic value of tuberculin.

Professor Bang reported 150 cases where the post-mortem examination revealed indications of tuberculosis through the use of tuberculin, and about 190 other cases where tuberculin had been used. Ninety-six per cent. of all those in which a reaction had been established proved to be tuberculous. He reported an instance where at the first test 10 per cent. of presumably healthy animals reacted; at the second year only one out of 107; and the succeeding spring two out of 122, showing a very rapid decrease in the number after the removal of the diseased ones. He stated that the greatest number of tuberculous animals were found where traffic was most active.

Hess, of Berne, Switzerland, reported that in his experience with tuberculin the animals sometimes exhibited severe symptoms, such as loss of appetite, great depression, and diminished yield of milk, the local swelling often lasting four and five days. Also, that acute tuberculosis was produced, and while he considered tuberculin an excellent diagnostic material, that there was great danger of producing acute miliary tuberculosis.

Professor Nocard, referring to the experiences in France, Saxony, Berlin, and Copenhagen, pointed to the fact that tuberculosis was generally contracted during adult life, and that heredity plays a very insignificant part in its spread. The Paris slaughter-houses exhibited few cases of tuberculosis among calves, while as high as 20 to 25 per cent. of the cows proved diseased. He considered tuberculin the best test, and had rarely seen a falling off in milk production after its use. He recommended all suspected animals to be tested, and those giving a reaction to be isolated from the healthy ones. In 2000 cases tested for tuberculin he had witnessed but two cases of generalized tuberculosis.

President Dammann, summing up the results of the discussion, stated: "The essayists are agreed that tuberculin is a most valuable diagnostic agent. The number of failures with it is practically unimportant. The greater number of those present do not share Hess's view of acute tuberculosis after inoculation, and therefore considered his warning unnecessary." —Ed.]

